# Predictors of the first cardiovascular event in patients with systemic lupus erythematosus - a prospective cohort study

**DOI:** 10.1186/ar2878

**Published:** 2009-12-10

**Authors:** Johanna Gustafsson, Iva Gunnarsson, Ola Börjesson, Susanne Pettersson, Sonia Möller, Guo-Zhong Fei, Kerstin Elvin, Julia F Simard, Lars-Olof Hansson, Ingrid E Lundberg, Anders Larsson, Elisabet Svenungsson

**Affiliations:** 1 Rheumatology Unit, Department of Medicine Karolinska University Hospital, Solna, Karolinska Institutet, SE-171 76 Stockholm, Sweden; 2 Unit of Clinical Immunology, Department of Clinical Immunology and Transfusion Medicine, Karolinska University Hospital, Karolinska Institutet, SE-171 76 Stockholm, Sweden; 3 Clinical Epidemiology Unit, Karolinska Institutet, SE-171 76 Stockholm, Sweden; 4 Department of Laboratory Medicine, Karolinska University Hospital, Karolinska Institutet, SE-171 76 Stockholm, Sweden; 5 Department of Clinical Chemistry and Pharmacology, Akademiska Hospital, SE-751 85 Uppsala, Sweden

## Abstract

**Introduction:**

Cardiovascular disease (CVD) is a major cause of premature mortality among Systemic lupus erythematosus (SLE) patients. Many studies have measured and evaluated risk factors for premature subclinical atherosclerosis, but few studies are prospective and few have evaluated risk factors for hard endpoints, i.e. clinically important cardiovascular events (CVE). We investigated the impact of traditional and lupus associated risk factors for the first ever CVE in a longitudinal cohort of SLE patients.

**Methods:**

A total of 182 SLE patients (mean age 43.9 years) selected to be free of CVE were included. Cardiovascular and autoimmune biomarkers were measured on samples collected after overnight fasting at baseline. Clinical information was collected at baseline and at follow up. End point was the first ever CVE (ischemic heart, cerebrovascular or peripheral vascular disease or death due to CVD). Impact of baseline characteristics/biomarkers on the risk of having a first CVE was evaluated with Cox regression.

**Results:**

Follow up was 99.5% after a mean time of 8.3 years. Twenty-four patients (13%) had a first CVE. In age-adjusted Cox regression, any positive antiphospholipid antibody (aPL), elevated markers of endothelial activation (von Willebrand factor (vWf), soluble vascular cellular adhesion molecule-1 (sVCAM-1)) and fibrinogen predicted CVEs. Of SLE manifestations, arthritis, pleuritis and previous venous occlusion were positively associated with future CVEs while thrombocytopenia was negatively associated. Among traditional risk factors only age and smoking were significant predictors. In a multivariable Cox regression model age, any positive aPL, vWf and absence of thrombocytopenia were all predictors of the first CVE.

**Conclusions:**

In addition to age, positive aPL, biomarkers indicating increased endothelial cell activity/damage, and absence of thrombocytopenia were independent predictors of CVEs in this prospective study. Our results indicate that activation of the endothelium and the coagulation system are important features in SLE related CVD. Furthermore, we observed that the risk of CVEs seems to differ between subgroups of SLE patients.

## Introduction

Systemic lupus erythematosus (SLE) is a heterogeneous chronic systemic autoimmune disease, which mainly affects women (90%). As treatment for lupus itself has gradually improved, mortality rates have declined and cardiovascular co-morbidity has become a growing clinical problem. Circulatory diseases are today a leading cause of mortality among SLE patients [1,2].

Traditional coronary artery disease (CAD) risk factors are more prevalent among SLE patients than in the general population [3,4] but they do not alone account for the high incidence of premature cardiovascular disease (CVD) seen in SLE [5,6]. Additionally, several SLE associated risk factors have been identified such as pro-thrombotic antiphospholipid antibodies (aPL) [4,7,8], accelerated endothelial cell apoptosis and impaired repair of damaged endothelium [9,10], oxidized low density lipoprotein (LDL) [11], pro-inflammatory high density lipoprotein (HDL) [12], genetic susceptibility [13] and decreased endothelial binding of annexin V [14]. The role of these and other mechanisms for premature cardiovascular morbidity and mortality seen in SLE are presently under intense study by many research groups.

Prospective studies that evaluate both traditional and lupus associated risk factors for hard endpoints, that is, CVEs, are to date relatively few in SLE [8,15,16] and outnumbered by studies focused on subclinical atherosclerosis in these patients [4,17,18]. But, measurements of atherosclerosis are surrogate markers of CVD and given the complexity of SLE, accelerated atherosclerosis may not be the only biologically plausible connection to CVEs. Other factors in an immunologically active setting like SLE may influence the likelihood of CVEs. It is therefore important to perform longitudinal studies in well-characterized SLE patients and to use hard endpoints such as myocardial infarction and stroke.

In a single center cohort of SLE patients, we selected patients free from clinical CVD and investigated the impact of traditional CAD risk factors, lupus associated biomarkers and clinical manifestations/features on the risk of presenting with a first ever CVE during eight years of follow-up.

## Materials and methods

### Patients

All SLE patients at the Department of Rheumatology, Karolinska University Hospital who fulfilled four or more of the 1982 revised American College of Rheumatology Criteria for classification of SLE [19] during the inclusion period (1995-99) were asked to participate. A total of 182 of 208 (87.5%) participants were free of previous CVEs and were included in this study. 94% of the patients were European Caucasians and six percent were of Asian origin.

At follow-up (2004-2007) living patients were reinvestigated in person when possible. If not, they were interviewed by telephone. Medical charts were reviewed for all patients and death certificates were collected from all deceased patients. Autopsy protocols were collected when available. The Local Ethics Committee of the Karolinska University Hospital approved the study. All patients gave informed consent to participate.

### Data collection

At baseline, patients were interviewed and examined by a rheumatologist who evaluated disease activity using Systemic Lupus Activity Measure (SLAM) [20] and organ damage with Systemic Lupus International Collaborating Clinics damage index (SLICC) [21]. A SLAM score >6 was considered as active disease [22]. Blood samples were taken after overnight fasting and laboratory examinations were performed blinded, either on fresh blood samples or after storage in -70°C. When stored samples were used (see Table 1), all samples were frozen and each investigation was carried out in one session.

**Table 1:** Baseline characteristics of patients

	All patients N = 182	Patients who developed CVE N = 24	Patients free of CVE N = 158	*P*
**Traditional risk factors**				
Age years	45 (31-53)	59 (49 to 66)	44 (30-52)	< 0.0001
Male gender	10%	9%	11%	
Smoking ever	51%	70%	46%	0.02
Hypertension	30%	42%	28%	
Systolic blood pressure (mmHg)*	125 (115 to 140)	140 (120 to 143)	120 (110 to 140)	0.03
Hypercholestrolemia	41%	54%	39%	
Total cholesterol (mmol/L)	4.9 (4.3 to 5.9)	5.7 (4.5 to 6.4)	4.9 (4.2 to 5.7)	0.05
LDL (mmol/L)	2.8 (2.3 to 3.5)	3.0 (2.7 to 4.0)	2.8 (2.2 to 3.5)	
HDL (mmol/L)	1.4 (1.1 to 1.7)	1.3 (1.0 to 1.9)	1.4 (1.1 to 1.7)	
Triglycerides (mmol/L)*	1.3 (1.0 to 1.9)	1.5 (1.2 to 2.0)	1.2 (0.9 to 1.9)	
ApoB/ApoA*†	0.5 (0.3 to 0.6)	0.6 (0.4 to 0.8)	0.5 (0.3 to 0.6)	
Diabetes	2%	0%	2%	
				
**Lupus manifestations**				
Disease duration	10 (4 to 18)	18 (10 to 24)	10 (4 to 17)	0.0005
Malar rash	58%	58%	58%	
Discoid rash	22%	22%	22%	
Photosensitivity	71%	50%	75%	0.01
Oral ulcers	29%	37%	27%	
Arthritis	84%	100%	82%	0.02
Pleuritis	38%	65%	34%	0.005
Pericarditis	18%	17%	22%	
Nephritis	30%	37%	29%	
Neurological disorder	12%	17%	11%	
Leucopenia	56%	57%	46%	
Thrombocytopenia	21%	4%	24%	0.03
Previous venous occlusion	12%	29%	9%	0.004
SLICC >1	53%	79%	49%	0.006
SLAM >6 at baseline	45%	50%	44%	
				
**Medication**				
Months on steroid treatment*	35 (3 to 118)	54 (1 to 123)	31 (3 to119)	
Cyclofosfamide treatment ever	15%	8%	16%	
Azathioprine at baseline	38%	4%	33%	
Chloroquine/Hydroxychloroquine at baseline	29%	21%	30%	
Warfarin at baseline	9%	13%	8%	
ASA at baseline	19%	25%	18%	
Statins at baseline	0.6%	0%	0.7%	
				
**Autoantibodies against**				
dsDNA	36%	33%	36%	
CL IgG	47%	58%	46%	
CL IgM	15%	21%	14%	
β_2_GP-1 IgG†	36%	54%	34%	0.05
Lupus anticoagulant	21%	25%	20%	
any PL	65%	83%	63%	0.05
SSA	43%	25%	46%	
SSB	24%	13%	25%	
Sm	11%	8%	12%	
RNP	11%	14%	11%	
				
**Markers of systemic inflammation**				
hs CRP (mg/L)*†	1.9 (0.7 to 5.1)	4.9 (1.7 to 23.5)	1.7 (0.6 to 4.1)	0.0002
Fibrinogen (g/L)*†	3.4 (2.9 to 4.5)	4.6 (3.3 to 5.3)	3.2 (2.8 to 4.3)	0.002
α-1 antitrypsine (g/L)†	1.5 (1.3 to 1.7)	1.8 (1.4 to 2.2)	1.5 (1.3 to 1.7	0.002
SAA (mg/L)*†	5.0 (2.5 to 10.9)	9.3 (5.1 to 26.8)	4.5 (2.3 to 9.3	0.004
IL 6 (ng/L)*†	3.3 (2.0 to 6.9)	5.3 (2.7 to 10.9)	3.1 (1.9 to 6.4)	0.03
C3 (g/L)†	1.0 (0.8 to 1.2)	1.0 (0.9 to 1.3)	1.0 (0.8 to 1.2)	
C3d (mg/L)†	11.8 (9.6 to 14.7)	12.9 (10.5 to 15.9)	11.6 (9.5 to 14.5)	
C4 (g/L)†	0.1 (0.1 to 0.2)	0.1 (0.09 to 1.4)	0.1 (0.1 to 0.2)	
				
**Markers of endothelial activation**				
sVCAM-1 (ng/L)*†	306 (252 to 385)	373 (303 to 503)	302 (249 to 375)	0.0009
von Willebrand factor %*†	117 (61 to 166)	157 (114 to 232)	112 (59 to 158)	0.001
				
**Markers of renal damage**				
Creatinine (μmol/L)*	82 (73 to 94)	87 (76 to 107)	81 (72 to 93)	0.01
MDRD ml/min/1.73 m^2^	68 (57 to 80)	59 (45 to 71)	69 (58 to 81)	0.004
Blood urea nitrogen mmol/L†	5.7 (4.7 to 7.2)	6.5 (5.3 to 9.9)	5.6 (4.6 to 6.8)	0.01
Pathologic urine	23%	25%	22%	
				
**Other biomarkers**				
Albumin (g/L)	45 (41 to 48)	41(37 to 45)	45 (42 to 48)	0.002
Homocystein (μmol/L)*†	12.3 (10.0 to 15.6)	12.1 (10.4 to 17.9)	12.4 (9.8 to 15.4)	

Distributions are given as % or median (interquartile range). *P *values, not adjusted for age, ≤ 0.05 are presented. * indicate not normally distributed variables. † indicate that analyses were done on frozen samples. Hypertension was defined as a systolic blood pressure >140 mm Hg and/or a diastolic blood pressure >90 mmHg and/or present treatment for hypertension. Hypercholesterolemia was defined as a total cholesterol level >5.2 mmol/L.

β_2_GP-1 = beta_2_glykoprotein-1; any PL = positive antibody test against CL IgG or IgM, β_2_GP-1 IgG or a positive lupus anticoagulant test; ApoB/ApoA = apolipoprotein B/A. Disease manifestations were defined according to the 1982 revised American College of Rheumatology (ACR) criteria for classification of SLE [19]; C = Complement factor; C3d = Complement factor 3 degradation products; CL = cardiolipin; dsDNA = doublestranded DNA; HDL = high density lipoprotein; hsCRP = high sensitivity C reactive protein; IL-6 = interleukin 6; LDL = low density lipoprotein; MDRD = Estimated glomerular filtration rate using the Modification of Diet in Renal Disease (MDRD) formula [51]. Pathologic urine as defined by SLAM [20]; PL = phospholipid; RNP = r ibonucleoprotein; SAA = serum amyloid A; SLAM = a measure of disease activity [20]; SLICC = a measure of cumulative disease damage [21], Sm = Smith; SSA = Sjogrens syndrome A;

SSB = Sjogrens syndrome B; sVCAM-1 = soluble vascular cell adhesion molecule 1.

Pathologic urine as defined by SLAM [20].

### Outcome measures of CVE

CVE were defined as: 1) Ischemic heart disease (IHD): myocardial infarction (MI, confirmed by electrocardiography and a rise in plasma creatine kinase, muscle and brain fraction (CK-MB) or troponin T) or angina pectoris (confirmed by exercise stress test).

2) Ischemic cerebrovascular disease (ICVD): cerebral infarction (confirmed by computer tomography) or transitory ischemic attacks (TIA, defined as transient focal symptoms from the brain or retina with a maximum duration of 24 hours).

3) Ischemic peripheral vascular disease (IPVD): intermittent claudication or peripheral arterial thrombosis/embolus (confirmed by angiogram or Doppler flow studies).

4) Death due to ischemic vascular disease: death due to myocardial infarction, heart failure, sudden death, cerebral infarction, generalized atherosclerosis as stated by death certificate.

### Laboratory Methods

High sensitivity C-reactive protein (CRP), α-1 antitrypsine, fibrinogen and Serum Amyloid A (SAA) were measured using BN ProSpec System (Dade Behring, Deerfield, IL, USA). C3 and C4 were analyzed using IMMAGE™ and C3d using an ARRAY™ system (both instruments are from Beckman Coulter, Brea, CA, USA). Albumin, apolipoprotein A1, apolipoprotein B and homocysteine were measured on an Architect Ci8200 analyzer (Abbott Laboratories, Abbott Park, IL, USA).

Enzyme-linked immunosorbent assays (ELISA) were used to measure Vascular cell adhesion molecule (VCAM-1, DY809, R&D Systems, Minneapolis, MN, USA), vWF (antisera from Dako (Glostrup, Denmark) calibrated against Liatest (Diagnostica Stago, Asnieres, France), intra-assay coefficient of variation were <7%) and Interleukin 6 (IL-6, high sensitive ELISA, HS600, R&D Systems, Minneapolis, MN, USA).

Antinuclear antibodies (ANA) were analysed by immunofluorescence on sections of rat liver and HEp-2 cells (Immunoconcepts, Sacramento, CA, USA) and auto-antibodies to Sjögren's syndrome A and B (SSA, SSB), Smith (Sm) and Ribonucloprotein (RNP) using ANA-profile ELISA (Pharmacia Diagnostics, Uppsala, Sweden), Innolia Immunoblot (Innogenetics, N.U. Ghent, Belgium), and Auto Immunodiffusion (Immunoconcepts, Sacramento, CA, USA). Anti-double-stranded DNA (dsDNA) antibodies were determined by IFL using *Crithidia lucillae *kinetoplast assay (Immunoconcepts, Sacramento, CA, USA). Anticardiolipin antibodies (aCL) were measured by ELISA using ethanol fixed cardiolipin (Sigma-Aldrich, St Louis, MO, USA) and HRP-conjugated rabbit immunoglobulins against human IgG, respectively IgM (Dako, Glostrup, Denmark). The levels of positivity were calibrated against Harris standard (Louisville, LAPL-GM-001). Low aCL level corresponded to 10-20, medium level to 20-80 and high level to >80 GPL/MPL Units. The cut off values for positive aCL was calculated to be at least the 95^th ^percentile of healthy blood donors. Autoantibodies to β_2_-glycoprotein1 (β_2_GP1, IgG) were analysed by ELISA (Orgentec, Mainz, Germany). Positive cut-off level was used according to the manufacturer's descriptions. Borderline results were regarded as negative. Lupus anticoagulant (LAC) was determined using a modified Dilute Russel Viper Venom method, (Biopool, Umea, Sweden) using Bioclot LAC.

### Statistics

Patient characteristics were summarized for all patients and by CVE occurrence and were compared using ANOVA, Mann-Whitney U-test and X^2 ^test as needed. Log transformation was used for skewed continuous variables. Hazard ratios (HR) and 95% confidence intervals (95% CI) for the first CVE were calculated in uni- and multivariable-adjusted Cox regression models for baseline factors.

Variables were first sorted into functional groups (traditional risk factors, lupus manifestations etc. see Table 1 and 2). After adjusting for age, the variables within each group, which were representative and most significantly associated with future CVEs were entered into a multivariable-adjusted model. Because of a limited number of first CVEs, we restricted the number of variables in the multivariable-adjusted model to four. Calculations were done using JMP software (SAS Institute, Carey, North Carolina, USA). A *P *value < 0.05 was considered statistically significant.

**Table 2:** Baseline predictors of the first ever cardiovascular event, page-adjusted Cox regression models

	Hazard Ratio	95% Confidence interval	*P*
**Traditional risk factors**			
Age univariate	2.28*	1.67 to 2.10	< 0.0001
	Age adjusted		
Male gender	0.71	0.11 to 2.41	
Smoking, ever	2.62	1.11 to 7.03	0.03
Hypertension	0.72	0.30 to 1.70	
Systolic blood pressure	0.78	0.52 to 1.18	
Hypercholesterolemia	1.37	0.61 to 3.14	
Total cholesterol	1.19	0.81 to 1.74	
LDL	1.23	0.80 to 1.89	
HDL	0.77	0.34 to 1.62	
Triglycerides†	1.21	0.86 to 1.69	
ApoB/ApoA†	1.36	0.88 to 2.10	
Diabetes	‡	‡	0.02
			
**Manifestations of Lupus**			
Disease duration	1.28	0.98 to 1.68	
Malar rash	1.09	0.72 to 1.67	
Discoid lupus	0.80	0.45 to 1.26	
Photosensitivity	0.69	0.46 to 1.04	
Oral ulcers	1.40	0.90 to 2.14	
Arthritis	§	§	0.002
Pleuritis	1.60	1.02 to 2.46	0.04
Pericarditis	1.35	0.77 to 2.16	
Nephritis	1.31	0.85 to 1.97	
Leukopenia	0.94	0.62 to 1.42	
Thrombocytpenia	0.37	0.09 to 0.83	0.009
Neurologic disorder	0.78	0.48 to 1.44	
Previous venous occlusion	1.88	1.16 to 2.90	0.01
SLICC>1	1.91	0.74 to 5.97	
SLAM>6 at baseline	1.25	0.83 to 1.87	
			
**Medications**			
Months on steroid treatment	1.00	0.68 to 1.48	
Cyclofosfamide treatment ever	0.60	0.01 to 2.03	
Azathioprine at baseline	0.51	0.03 to 2.47	
Chloroquine/Hydroxychloroquine at baseline	1.01	0.32 to 2.67	
Warfarin at baseline	1.45	0.34 to 4.27	
ASA at baseline	1.54	0.55 to 3.70	
			
**Autoantibodies against**			
dsDNA	1.41	0.57 to 3.25	
CL IgG	2.57	1.13 to 6.13	0.02
CL IgM	1.35	0.45 to 3.41	
β_2_GP1 IgG	2.57	1.13 to 5.99	0.02
Lupus anticoagulant	1.10	0.40 to 2.63	
Any aPL	4.90	1.76 to 17.72	0.002
SSA	0.66	0.39 to 1.03	
SSB	0.57	0.27 to 1.00	0.05
Sm	1.21	0.48 to 2.29	
RNP	1.60	0.76 to 2.87	
			
**Markers of systemic inflammation**			
hs CRP†	1.36	0.85 to 2.18	
Fibrinogen†	1.72	1.10 to 2.68	0.02
α-1 antitrypsine	1.49	1.01 to 2.18	
SAA†	1.36	0.88 to 2.09	
IL 6†	1.26	0.87 to 1.82	
C3	0.92	0.63 to 1.35	
C3d	1.38	0.85 to 2.22	
C4	0.82	0.56 to 1.18	
			
**Markers of endothelial activation**			
sVCAM-1†	1.78	1.20 to 2.65	0.005
von Willebrand factor†	2.05	1.23 to 3.42	0.004
			
**Markers of renal involvement**			
Creatinine†	1.17	0.74 to 1.85	
MDRD	1.07	0.57 to 2.00	
Blood urea nitrogen	1.15	0.75 to 1.77	
Pathological urine	2.4	0.8 to 6.1	
			
**Other biomarkers**			
Albumin(g/l)	0.56	0.41 to 0.78	0.002
Homocystein (μmol/l)†	0.79	0.52 to 1.21	

Calculations were done using age-adjusted Cox regression. For continuous variables Hazard Ratio (HR) is given as risk per standard deviation, *For age HR is calculated per 10 years (HR/10 years). †calculations were done on log transformed values. *P *values ≤ 0.05 are presented. ‡ Infinite coefficient, due to no AE in the diabetes group. §Infinite coefficient, due to all AE in the arthritis group, confidence interval could thus not be determined for these two variables. Hypertension was defined as a systolic blood pressure >140 mm Hg, and/or a diastolic blood pressure >90 mmHg and/or present treatment for hypertension. Hypercholesterolemia was defined as a total cholesterol level >5.2 mmol/L.

β_2_GP-1 = beta2glykoprotein-1; β_2_GP-1 IgG or a positive lupus anticoagulant test;

any PL = positive antibody test against CL IgG or IgM; ApoB/ApoA = apolipoproteinB/A. Disease manifestations were defined according to the 1982 revised American College of Rheumatology (ACR) criteria for classification of SLE [19]; C = Complement factor; C3d = Complement factor 3 degradation products; CL = cardiolipin; dsDNA = doublestranded DNA; HDL = high density lipoprotein; hsCRP = high sensitivity C reactive protein; IL-6 = interleukin 6; LDL = low density lipoprotein;

MDRD = Estimated glomerular filtration rate using the Modification of Diet in Renal Disease (MDRD) formula [51]. Pathologic urine as defined by SLAM [20]; PL = phospholipid; RNP = ribonucleoprotein; SAA = serum amyloid A; SLAM = a measure of disease activity [20]; SLICC = a measure of cumulative disease damage [21]; Sm = Smith; SSA = Sjogrens syndrome A; SSB = Sjogrens syndrome B; sVCAM-1 = soluble vascular cell adhesion molecule 1.

## Results

### Baseline data and clinical manifestations

As expected, patients who developed a first CVE were older, more likely to have ever smoked, have higher total cholesterol levels and to have higher systolic blood pressure. Furthermore, they had a longer mean SLE duration, were more likely to have had previous venous occlusion, pleuritis, arthritis and leucopenia but were less likely to have photosensitivity or thrombocytopenia. Several markers of systemic inflammation, endothelial activation and renal damage, together with aPL, were elevated at baseline in patients who developed a first CVE (Table 1).

### Cardiovascular events

Surviving patients were reinvestigated after a mean time of 8.3 ± 1.2 years. A total 132 patients were examined in person, and 34 through a structured telephone interview. Fifteen patients were deceased and their death certificates were investigated. Five autopsies had been performed and all protocols were reviewed. Medical files were reviewed for all with the exception of one patient who was unwilling to participate at follow up, thus her history of CVE could not be determined.

Twenty-four new CVEs occurred, three of which were fatal (two heart failures, one atherosclerosis according to death certificates) (Table 3).

**Table 3:** Age at and type of first cardiovascular event

Type of cardiovascular event	Number of events	Median age at onset	Age range
All cardiovascular events	24	64.4	40.6 to 85.1
Ischemic heart disease	10	60.8	40.6 to 85.1
Ischemic cerebrovascular disease	6	65.7	49.9 to 83.9
Ischemic peripheral vascular disease	5	55.1	44.1 to 82.4
Death due to cardiovascular disease	3	64.7	58.1 to 68.6

Ischemic heart disease = myocardial infarction or angina pectoris, ischemic cerebrovascular disease = cerebral infarction or transitory ischemic attacks, ischemic peripheral vascular disease = intermittent claudication or peripheral arterial thrombosis/embolus, death due to ischemic vascular disease = death due to myocardial infarction, heart failure, sudden death, cerebral infarction, generalized atherosclerosis as stated by death certificate.

### Prospective evaluation of risk factors for CVEs

As expected, age was a strong predictor of the first CVE, we therefore adjusted all measurements for age. Thereafter, only smoking of the traditional risk factors remained as a predictor of CVEs. Of lupus manifestations arthritis, pleuritis, previous venous occlusion and absence of thrombocytopenia were associated with first CVEs. Among biomarkers aPL (aCL IgG, β GP1 IgG and any aPL), fibrinogen, markers of endothelial activation (sVCAM-1 and vWf), low levels of plasma albumin, and absence of SSB antibodies remained as predictors of the first CVE. All patients with a first CVE had arthritis, and none had diabetes, therefore these variables could not be further evaluated (Table 2). In a multivariable-adjusted model age, any positive aPL, and vWf were positively associated with CVEs, while thrombocytopenia was inversely associated (Table 4). In an alternative model we exchanged vWf for sVCAM-1. sVCAM remained in this model, together with the other variables in Table 4, as an independent predictor of CVEs (Relative Risk for sVCAM-1 = 2.11, 95%CI (1.24 to 3.58), *P *= 0.005).

**Table 4:** Baseline predictors of the first ever cardiovascular event, multivariable-adjusted Cox regression

	Hazard Ratio	Confidence interval	*P*
Age*	2.39	1.71 to 3.32	< 0.0001
Thrombocytopenia	0.35	0.08 to 0.77	0.005
Any aPL	4.23	1.56 to 14.83	0.003
von Willebrand factor†	1.97	1.16 to 3.33	0.01

Calculations were done using multivariable-adjusted Cox regression. *For age hazard ratio is calculated per 10 years (HR/10 years), for von Willebrand factor hazard ratio is given as risk per standard deviation, †calculations were done on log transformed values.

## Discussion

This is, to our knowledge, the first study to investigate predictors of the first ever CVE in SLE and a novel observation is that high levels of endothelial markers precede these events. We also identified SLE manifestations and can confirm [8] occurrence of aPLs as predictors of first CVEs. It is of note that, with the exception of age and smoking, traditional CAD risk factors did not explain CVEs in this study.

In healthy individuals high circulating levels of vWf predicted CVEs in several studies [23,24], but in contrast to this study, significance did not remain after adjustment for other CAD risk factors [24,25]. Myocardial infarctions are common in conditions with high levels of vWf. Plasma levels rise during acute coronary syndromes and high levels predict less favorable long time outcome in patients with established CVD, as recently reviewed by Spiel et.al [26]. Inflammatory cytokines can cause release of vWf from endothelial cells into the circulation where vWf has pro-thrombotic effects as it promotes aggregation of platelets [26]. SLE patients with subclinical atherosclerosis have increased vWf activity in the circulation [27] and vWf was furthermore induced by IgG from patients with SLE and thrombotic diseases [28] and by anti DNA antibodies in experimental studies [29]. The high activity of vWf in lupus [27,30] may thus be attributable to both pro-inflammatory cytokines and to autoantibodies, and could directly contribute to the high incidence of CVE in SLE.

VCAM-1, an endothelial adhesion molecule is shed into the circulation when the endothelium is activated. In SLE, levels of sVCAM-1 are elevated and correlate with disease activity [31], renal manifestations [32] and aPL [33]. We have recently demonstrated that after adjustment for nephritis, high levels of sVCAM-1 discriminated SLE patients with manifest CVD from SLE patients free of CVD and population controls [34]. The present study extends these findings as we can report that high levels of sVCAM-1 predict future CVE, independently of aPLs. VCAM-1 has also been reported to predict poor long-term outcome in other patient groups at risk of CVD, that is, in hemodialysis patients [35], in patients with diabetic nephropathy [36] and in patients with manifest CAD [37]. Both vWf and VCAM-1 are markers of endothelial activation. Other studies have reported impaired endothelial function as measured by flow mediated dilatation [38] and that increased numbers of circulating apoptotic endothelial cells are present in SLE patients [39]. Taken together many studies report that endothelial perturbation occurs in SLE, and our results suggest that it precedes the first CVE.

In the general population, systemic inflammatory activity, often measured by CRP, is a well-documented risk factor for CVD. A similar role for fibrinogen, SAA and IL-6, has also been reported [40]. At baseline elevation of several inflammatory markers were more prevalent among patients who experienced their first CVE during follow-up, but after adjustment for age, only fibrinogen remained as a significant predictor. Fibrinogen is an important clotting protein, and high levels have been reported in SLE patients with a history of thrombotic disease [41]. CRP has previously been identified as a risk factor for arterial disease in SLE, but we were not able to replicate those results [8].

Patients with a positive test for any aPL had a higher risk of CVEs confirming previous findings by Toloza et.al [8]. Positive IgG titers of aCL, or β_2_GP1, were predictive of CVE. However, IgM aCL and LAC were not. Any positive aPL were more common in this study (65%) than reported in most [7,33,42] lupus cohorts possibly indicating a low cut-off level at our laboratory in the mid 90's when baseline data were collected. It is therefore interesting to note that any aPL were only predictive of CVEs when we included the cut-off for positive tests. With a cut-off at medium titer, more in accordance with the definition of the anti-phospholipid syndrome (APS) [43], baseline aPLs were no longer significantly associated with future CVE (data not shown). This may be explained by limited power, as few patients had medium/high titer aPL, and by the fact that these patients were more often on anti-coagulation treatment. Nevertheless, our results suggest that also low titer aPL adds to the risk of CVEs in SLE patients.

We noted that subsets of patients with different lupus manifestations seemed to have a differentiated risk of CVE. Arthritis and pleuritis are SLE manifestations, often associated with a more pronounced acute phase response [15,44], and patients with these symptoms had a higher risk of developing a first CVE, as also reported by Mok et al [16]. Patients with a previous episode of venous occlusion, a major manifestation of APS [43], were more likely to present with a first CVE. They were also more likely to have any positive aPL (Odds Ratio = 12.5, 95% CI (1.6 to 95.7), *P *= 0.0005). The observation that patients with previous thrombocytopenia, also associated with APS [43], were at lower risk of CVEs is new. Previous studies have demonstrated that thrombocytopenia is often associated with auto antibodies to platelet glycoproteins, but these antibodies have not been linked to thrombosis [45]. Hypothetically a low platelet count could confer a protective anti-coagulant effect, but this would need to be tested in larger samples. Another novel observation is that patients with photosensitivity and SSA/SSB antibodies, which often occur together, may be at lower CVD risk as compared to other SLE patients. Taken together, this study suggests that subgroups of SLE patients are not affected with the same high risk of CVEs. Larger cohort studies are needed to confirm these observations.

Besides age, smoking was the only traditional CVD risk factor which predicted CVEs in this study, this is in line with the results of Toloza et al [8] Lipid derangements including hypercholesterolemia, elevated ApoB/ApoA ratio, low HDL and hypertriglyceridemia, did not remain statistically significant after adjustment for age. Earlier studies are inconsistent with regard to the impact of blood lipids on CVD in SLE. Some have reported an association between manifest CVD and hypercholesterolemia [46,47], hypertriglyceridemia [48,49] or low HDL [4,16] whereas this study and the prospective study from the LUMINA cohort did not find an association [8].

After adjusting for age, a lower estimated glomerular filtration rate at baseline was not predictive of presenting with a first CVE in this study. Hypoalbuminemia, positively associated both with markers of systemic inflammation and with loss of renal function (data not shown), was however a strong predictor. It was not entered into the multivariable-adjusted model because we regarded it as a proxy for both systemic inflammation and for renal disease. Its significance may however be useful since albumin measurements are inexpensive and commonly used in clinical practice.

The relatively small number of patients and events in the present study are limitations, which call for some caution, in particular with regard to subgroup analysis. It may also explain the lack of association with traditional CAD risk factors. The great majority of our patients were Caucasians of European origin, thus confirmation in other ethnic groups is needed before generalizing our results to SLE cohorts from other parts of the world. The detailed baseline information and the relatively long and almost complete follow-up including all deceased patients are strengths of the study. The fact that we only included the first ever CVE is a merit, but also a reason for the low number of events. Several patients eventually experienced additional events. Of note 9/24 patients (38%) who experienced a first CVE were dead when we closed the study in 2007. This figure further highlights the importance of conducting longitudinal prospective studies.

Due to the design of this study and the clinical definitions used for CVEs it was not possible to compare the incidence of CVEs in these lupus patients with the general population. However, other studies have shown that patients with SLE have a marked increased risk of CVD as compared to the general population [2,15,50].

## Conclusions

In conclusion this prospective study is the first to investigate lupus associated and CAD risk factors for the first ever CVE in patients with SLE. In addition to age, positive aPL, biomarkers indicating increased endothelial cell activity/damage, and absence of thrombocytopenia were independent predictors of CVEs. Our results indicate that activation of the endothelium and the coagulation system are important features in SLE related CVD. Furthermore, the risk of CVEs seems to differ between subgroups of SLE patients. How these findings influence and interact with the presence of premature atherosclerosis, not measured in this study, remains to be determined.

## Abbreviations

aCL: anticardiolipin antibodies; ANA: antinuclear antibodies; ApoB/ApoA: apolipoproteinB/A; APS: anti-phospholipid syndrome; aPL: antiphospholipid antibody; β_2_GP1: β_2_-glycoprotein1; C: complement factor; C3d: complement factor 3 degradation products; CAD: coronary artery disease; CI: confidence interval; CVD: cardiovascular disease; CVE: cardiovascular event; dsDNA: anti-double-stranded DNA; ELISA: enzyme-linked immunosorbent assays; GPL/MPL: standardized unit used for measuring anticardiolipin antibodies (one unit approximated to 1 g of immunoglobulin G (IgG) (for GPL) or IgM (for MPL) anticardiolipin antibody purified from 1 mL of serum.); HDL: high density lipoprotein; HR: hazard ratio; HRP: horseradish peroxidase; hsCRP: high sensitivity C reactive protein; ICVD: ischemic cerebrovascular disease; IFL: immunofluorescence; IHD: ischemic heart disease; IL-6: interleukin 6; IPVD: ischemic peripheral vascular disease; LAC: lupus anticoagulant; LDL: low density lipoprotein; MDRD: estimated glomerular filtration rate using the Modification of Diet in Renal Disease formula; RNP: ribonucleoprotein; SAA: serum amyloid A; SLAM: systemic lupus activity measure; SLE: systemic lupus erythematosus; SLICC: systemic lupus international collaborating clinics damage index; Sm: Smith; SSA/SSB: Sjögren's syndrome A and B; sVCAM-1: soluble vascular cell adhesion molecule 1; vWf: von Willebrand factor.

## Competing interests

The authors declare that they have no competing interests.

## Authors' contributions

JG acquired, analyzed and interpreted the data and drafted the manuscript. IG OB, SP, SM and GZF acquired the data, KE coordinated and acquired the analysis of autoantibodies, JFS analyzed the data and drafted the manuscript, LOH and AL coordinated and acquired the laboratory data, IEL acquired the data ES conceived and designed the study, acquired, analyzed and interpreted the data and drafted the manuscript. All authors reviewed and approved the final manuscript.

## References

[B1] Bernatsky S Boivin JF Joseph L Manzi S Ginzler E Gladman DD Urowitz M Fortin PR Petri M Barr S Gordon C Bae SC Isenberg D Zoma A Aranow C Dooley MA Nived O Sturfelt G Steinsson K Alarcon G Senecal JL Zummer M Hanly J Ensworth S Pope J Edworthy S Rahman A Sibley J El-Gabalawy H McCarthy T Mortality in systemic lupus erythematosus Arthritis Rheum 2006 54 2550 2557 10.1002/art.21955 16868977

[B2] Bjornadal L Yin L Granath F Klareskog L Ekbom A Cardiovascular disease a hazard despite improved prognosis in patients with systemic lupus erythematosus: results from a Swedish population based study 1964-95 J Rheumatol 2004 31 713 719 15088296

[B3] Bruce IN Urowitz MB Gladman DD Ibanez D Steiner G Risk factors for coronary heart disease in women with systemic lupus erythematosus: the Toronto Risk Factor Study Arthritis Rheum 2003 48 3159 3167 10.1002/art.11296 14613278

[B4] Svenungsson E Jensen-Urstad K Heimburger M Silveira A Hamsten A de Faire U Witztum JL Frostegard J Risk factors for cardiovascular disease in systemic lupus erythematosus Circulation 2001 104 1887 1893 10.1161/hc4101.097518 11602489

[B5] Bessant R Hingorani A Patel L MacGregor A Isenberg DA Rahman A Risk of coronary heart disease and stroke in a large British cohort of patients with systemic lupus erythematosus Rheumatology (Oxford) 2004 43 924 929 10.1093/rheumatology/keh213 15150430

[B6] Esdaile JM Abrahamowicz M Grodzicky T Li Y Panaritis C du Berger R Cote R Grover SA Fortin PR Clarke AE Senecal JL Traditional Framingham risk factors fail to fully account for accelerated atherosclerosis in systemic lupus erythematosus Arthritis Rheum 2001 44 2331 2337 10.1002/1529-0131(200110)44:10<2331::AID-ART395>3.0.CO;2-I 11665973

[B7] Nojima J Masuda Y Iwatani Y Kuratsune H Watanabe Y Suehisa E Takano T Hidaka Y Kanakura Y Arteriosclerosis obliterans associated with anti-cardiolipin antibody/beta2-glycoprotein I antibodies as a strong risk factor for ischaemic heart disease in patients with systemic lupus erythematosus Rheumatology (Oxford) 2008 47 684 689 10.1093/rheumatology/ken124 18375400

[B8] Toloza SM Uribe AG McGwin G Jr Alarcon GS Fessler BJ Bastian HM Vila LM Wu R Shoenfeld Y Roseman JM Reveille JD Systemic lupus erythematosus in a multiethnic US cohort (LUMINA). XXIII. Baseline predictors of vascular events Arthritis Rheum 2004 50 3947 3957 15593203 10.1002/art.20622

[B9] Denny MF Thacker S Mehta H Somers EC Dodick T Barrat FJ McCune WJ Kaplan MJ Interferon-alpha promotes abnormal vasculogenesis in lupus: a potential pathway for premature atherosclerosis Blood 2007 110 2907 2915 10.1182/blood-2007-05-089086 17638846 PMC2018671

[B10] Westerweel PE Luijten RK Hoefer IE Koomans HA Derksen RH Verhaar MC Haematopoietic and endothelial progenitor cells are deficient in quiescent systemic lupus erythematosus Ann Rheum Dis 2007 66 865 870 10.1136/ard.2006.065631 17329307 PMC1955125

[B11] Frostegard J Nilsson J Haegerstrand A Hamsten A Wigzell H Gidlund M Oxidized low density lipoprotein induces differentiation and adhesion of human monocytes and the monocytic cell line U937 Proc Natl Acad Sci USA 1990 87 904 908 10.1073/pnas.87.3.904 2300583 PMC53377

[B12] McMahon M Grossman J Skaggs B Fitzgerald J Sahakian L Ragavendra N Charles-Schoeman C Watson K Wong WK Volkmann E Chen W Gorn A Karpouzas G Weisman M Wallace DJ Hahn BH Dysfunctional proinflammatory high-density lipoproteins confer increased risk of atherosclerosis in women with systemic lupus erythematosus Arthritis Rheum 2009 60 2428 2437 10.1002/art.24677 19644959 PMC2753974

[B13] Svenungsson E Gustafsson J Leonard D Sandling J Gunnarsson I Nordmark G Jonsen A Bengtsson AA Sturfelt G Rantapaa-Dahlqvist S Elvin K Sundin U Garnier S Simard JF Sigurdsson S Padyukov L Syvanen AC Ronnblom L A STAT4 risk allele is associated with ischemic cerebrovascular events and antiphospholipid antibodies in Systemic Lupus Erythematosus Ann Rheum Dis 2009 doi:10.1136/ard.2009.115535 19762360 10.1136/ard.2009.115535

[B14] Cederholm A Svenungsson E Jensen-Urstad K Trollmo C Ulfgren AK Swedenborg J Fei GZ Frostegard J Decreased binding of annexin v to endothelial cells: a potential mechanism in atherothrombosis of patients with systemic lupus erythematosus Arterioscler Thromb Vasc Biol 2005 25 198 203 15539620 10.1161/01.ATV.0000150415.18759.36

[B15] Jonsson H Nived O Sturfelt G Outcome in systemic lupus erythematosus: a prospective study of patients from a defined population Medicine (Baltimore) 1989 68 141 150 2785628

[B16] Mok CC Tang SS To CH Petri M Incidence and risk factors of thromboembolism in systemic lupus erythematosus: a comparison of three ethnic groups Arthritis Rheum 2005 52 2774 2782 10.1002/art.21224 16142761

[B17] Roman MJ Shanker BA Davis A Lockshin MD Sammaritano L Simantov R Crow MK Schwartz JE Paget SA Devereux RB Salmon JE Prevalence and correlates of accelerated atherosclerosis in systemic lupus erythematosus N Engl J Med 2003 349 2399 2406 10.1056/NEJMoa035471 14681505

[B18] Westerweel PE Luyten RK Koomans HA Derksen RH Verhaar MC Premature atherosclerotic cardiovascular disease in systemic lupus erythematosus Arthritis Rheum 2007 56 1384 1396 10.1002/art.22568 17469095

[B19] Tan EM Cohen AS Fries JF Masi AT McShane DJ Rothfield NF Schaller JG Talal N Winchester RJ The 1982 revised criteria for classification of systemic lupus erythematosus Arthritis Rheum 1982 25 1271 1277 10.1002/art.1780251101 7138600

[B20] Liang MH Socher SA Roberts WN Esdaile JM Measurement of systemic lupus erythematosus activity in clinical research Arthritis Rheum 1988 31 817 825 10.1002/art.1780310701 3293570

[B21] Gladman D Ginzler E Goldsmith C Fortin P Liang M Urowitz M Bacon P Bombardieri S Hanly J Hay E Isenberg D Jones J Kalunian K Maddison P Nived O Petri M Richter M Sanchez-Guerrero J Snaith M Sturfelt G Symmons D Zoma A The development and initial validation of the systemic lupus international collaborating clinics/American College of Rheumatology Damage index for Systemic lupus erythematosus Arthritis Rheum 1996 39 363 369 10.1002/art.1780390303 8607884

[B22] Manzi S Rairie JE Carpenter AB Kelly RH Jagarlapudi SP Sereika SM Medsger TA Jr Ramsey-Goldman R Sensitivity and specificity of plasma and urine complement split products as indicators of lupus disease activity Arthritis Rheum 1996 39 1178 1188 10.1002/art.1780390716 8670328

[B23] Morange PE Simon C Alessi MC Luc G Arveiler D Ferrieres J Amouyel P Evans A Ducimetiere P Juhan-Vague I Endothelial cell markers and the risk of coronary heart disease: the Prospective Epidemiological Study of Myocardial Infarction (PRIME) study Circulation 2004 109 1343 1348 10.1161/01.CIR.0000120705.55512.EC 15023872

[B24] Thogersen AM Jansson JH Boman K Nilsson TK Weinehall L Huhtasaari F Hallmans G High plasminogen activator inhibitor and tissue plasminogen activator levels in plasma precede a first acute myocardial infarction in both men and women: evidence for the fibrinolytic system as an independent primary risk factor Circulation 1998 98 2241 2247 9826309 10.1161/01.cir.98.21.2241

[B25] Whincup PH Danesh J Walker M Lennon L Thomson A Appleby P Rumley A Lowe GD von Willebrand factor and coronary heart disease: prospective study and meta-analysis Eur Heart J 2002 23 1764 1770 10.1053/euhj.2001.3237 12419296

[B26] Spiel AO Gilbert JC Jilma B von Willebrand factor in cardiovascular disease: focus on acute coronary syndromes Circulation 2008 117 1449 1459 10.1161/CIRCULATIONAHA.107.722827 18347221

[B27] de Leeuw K Freire B Smit AJ Bootsma H Kallenberg CG Bijl M Traditional and non-traditional risk factors contribute to the development of accelerated atherosclerosis in patients with systemic lupus erythematosus Lupus 2006 15 675 682 10.1177/0961203306069972 17120595

[B28] Lindsey NJ Dawson RA Henderson FI Greaves M Hughes P Stimulation of von Willebrand factor antigen release by immunoglobulin from thrombosis prone patients with systemic lupus erythematosus and the anti-phospholipid syndrome Br J Rheumatol 1993 32 123 126 10.1093/rheumatology/32.2.123 8428224

[B29] Lai KN Leung JC Lai KB Lai FM Wong KC Increased release of von Willebrand factor antigen from endothelial cells by anti-DNA autoantibodies Ann Rheum Dis 1996 55 57 62 10.1136/ard.55.1.57 8572736 PMC1010083

[B30] Ferro D Pittoni V Quintarelli C Basili S Saliola M Caroselli C Valesini G Violi F Coexistence of anti-phospholipid antibodies and endothelial perturbation in systemic lupus erythematosus patients with ongoing prothrombotic state Circulation 1997 95 1425 1432 9118509 10.1161/01.cir.95.6.1425

[B31] Spronk PE Bootsma H Huitema MG Limburg PC Kallenberg CG Levels of soluble VCAM-1, soluble ICAM-1, and soluble E-selectin during disease exacerbations in patients with systemic lupus erythematosus (SLE); a long term prospective study Clin Exp Immunol 1994 97 439 444 7521807 10.1111/j.1365-2249.1994.tb06107.x PMC1534867

[B32] Ikeda Y Fujimoto T Ameno M Shiiki H Dohi K Relationship between lupus nephritis activity and the serum level of soluble VCAM-1 Lupus 1998 7 347 354 10.1191/096120398678920172 9696139

[B33] Farzaneh-Far A Roman MJ Lockshin MD Devereux RB Paget SA Crow MK Davis A Sammaritano L Levine DM Salmon JE Relationship of antiphospholipid antibodies to cardiovascular manifestations of systemic lupus erythematosus Arthritis Rheum 2006 54 3918 3925 10.1002/art.22265 17133599

[B34] Svenungsson E Cederholm A Jensen-Urstad K Fei GZ de Faire U Frostegard J Endothelial function and markers of endothelial activation in relation to cardiovascular disease in systemic lupus erythematosus Scand J Rheumatol 2008 37 352 359 10.1080/03009740802007514 18666029

[B35] Papagianni A Dovas S Bantis C Belechri AM Kalovoulos M Dimitriadis C Efstratiadis G Alexopoulos E Memmos D Carotid atherosclerosis and endothelial cell adhesion molecules as predictors of long-term outcome in chronic hemodialysis patients Am J Nephrol 2008 28 265 274 10.1159/000110895 17989499

[B36] Wu T McGrath KC Death AK Cardiovascular disease in diabetic nephropathy patients: cell adhesion molecules as potential markers? Vasc Health Risk Manag 2005 1 309 316 10.2147/vhrm.2005.1.4.309 17315603 PMC1993958

[B37] Blankenberg S Rupprecht HJ Bickel C Peetz D Hafner G Tiret L Meyer J Circulating cell adhesion molecules and death in patients with coronary artery disease Circulation 2001 104 1336 1342 10.1161/hc3701.095949 11560847

[B38] Lima DS Sato EI Lima VC Miranda F Jr Hatta FH Brachial endothelial function is impaired in patients with systemic lupus erythematosus J Rheumatol 2002 29 292 297 11842823

[B39] Rajagopalan S Somers EC Brook RD Kehrer C Pfenninger D Lewis E Chakrabarti A Richardson BC Shelden E McCune WJ Kaplan MJ Endothelial cell apoptosis in systemic lupus erythematosus: a common pathway for abnormal vascular function and thrombosis propensity Blood 2004 103 3677 3683 10.1182/blood-2003-09-3198 14726373

[B40] Willerson JT Ridker PM Inflammation as a cardiovascular risk factor Circulation 2004 109 II2 II10 10.1161/01.CIR.0000129535.04194.38 15173056

[B41] Ames PR Alves J Pap AF Ramos P Khamashta MA Hughes GR Fibrinogen in systemic lupus erythematosus: more than an acute phase reactant? J Rheumatol 2000 27 1190 1195 10813286

[B42] Sturfelt G Eskilsson J Nived O Truedsson L S V Cardiovascular Disease in Systemic Lupus Erythematosus, A Study of 75 Patients from a Defined Population Medicine 1992 71 216 223 10.1097/00005792-199207000-00004 1518395

[B43] Miyakis S Lockshin MD Atsumi T Branch DW Brey RL Cervera R Derksen RH PG DEG Koike T Meroni PL Reber G Shoenfeld Y Tincani A Vlachoyiannopoulos PG Krilis SA International consensus statement on an update of the classification criteria for definite antiphospholipid syndrome (APS) J Thromb Haemost 2006 4 295 306 10.1111/j.1538-7836.2006.01753.x 16420554

[B44] Spronk PE ter Borg EJ Kallenberg CG Patients with systemic lupus erythematosus and Jaccoud's arthropathy: a clinical subset with an increased C reactive protein response? Ann Rheum Dis 1992 51 358 361 10.1136/ard.51.3.358 1575582 PMC1004661

[B45] Galli M Daldossi M Barbui T Anti-glycoprotein Ib/IX and IIb/IIIa antibodies in patients with antiphospholipid antibodies Thromb Haemost 1994 71 571 575 8091382

[B46] Manzi S Meilahn EN Rairie JE Conte CG Medsger TA Jr Jansen-McWilliams L D'Agostino RB Kuller LH Age-specific incidence rates of myocardial infarction and angina in women with systemic lupus erythematosus: comparison with the Framingham Study Am J Epidemiol 1997 145 408 415 9048514 10.1093/oxfordjournals.aje.a009122

[B47] Petri M Pereez-Gutthann S Spence D MC H Risk Factors for coronary Artery Disease in Patients With Systemic Lupus Erythematosus The American Journal of Medicine 1992 93 513 519 10.1016/0002-9343(92)90578-Y 1442853

[B48] Bessant R Duncan R Ambler G Swanton J Isenberg DA Gordon C Rahman A Prevalence of conventional and lupus-specific risk factors for cardiovascular disease in patients with systemic lupus erythematosus: A case-control study Arthritis Rheum 2006 55 892 899 10.1002/art.22343 17139666

[B49] Svenungsson E Fei GZ Jensen-Urstad K de Faire U Hamsten A Frostegard J TNF-alpha: a link between hypertriglyceridaemia and inflammation in SLE patients with cardiovascular disease Lupus 2003 12 454 461 10.1191/0961203303lu412oa 12873047

[B50] Hak AE Karlson EW Feskanich D Stampfer MJ Costenbader KH Systemic lupus erythematosus and the risk of cardiovascular disease: results from the nurses' health study Arthritis Rheum 2009 61 1396 1402 10.1002/art.24537 19790130 PMC2909444

[B51] Levey AS Bosch JP Lewis JB Greene T Rogers N Roth D A more accurate method to estimate glomerular filtration rate from serum creatinine: a new prediction equation. Modification of Diet in Renal Disease Study Group Ann Intern Med 1999 130 461 470 10075613 10.7326/0003-4819-130-6-199903160-00002

